# Effects of Paediatric Post-COVID-19 Condition on Physical Function and Daily Functioning: A Cross-Sectional Study

**DOI:** 10.3390/children12091216

**Published:** 2025-09-10

**Authors:** Aroia Goicoechea-Calvo, Roser Coll-Fernández, Natalia Navarro Expósito, Marc Colomer Giralt, Alba González-Aumatell, María Méndez-Hernández, Clara Carreras-Abad, Natàlia Pallarès Fontanet, Cristian Tebe Cordomi, M. J. Durà Mata, Carlos Rodrigo

**Affiliations:** 1 Department of Rehabilitation, Germans Trias i Pujol University Hospital, 08916 Badalona, Spain; rcollf.germanstrias@gencat.cat (R.C.-F.); nnavarroe.germanstrias@gencat.cat (N.N.E.); mcolomer.germanstrias@gencat.cat (M.C.G.); mjdura.germanstrias@gencat.cat (M.J.D.M.); 2 Department of Pediatrics, Obstetrics and Gynecology, Preventive Medicine and Public Health, Faculty of Medicine, Universitat Autònoma de Barcelona, 08193 Cerdanyola del Vallès, Spain; agonzalezau.germanstrias@gencat.cat (A.G.-A.); mjmendez.germanstrias@gencat.cat (M.M.-H.); claracarrerasa.germanstrias@gencat.cat (C.C.-A.); crodrigo.germanstrias@gencat.cat (C.R.); 3 Department of Pediatrics, Germans Trias i Pujol University Hospital, 08916 Badalona, Spain; 4 Germans Trias i Pujol Research Institute and Hospital (IGTP), 08916 Badalona, Spain; 5 Biostatistics Support and Research Unit, Germans Trias i Pujol Research Institute and Hospital (IGTP), 08916 Badalona, Spain; npallares@igtp.cat (N.P.F.); ctebe@igtp.cat (C.T.C.); 6 Department of Medicine, Universitat Autònoma de Barcelona, 08193 Cerdanyola del Vallès, Spain

**Keywords:** paediatric post-COVID-19 condition, long COVID, children, exercise capacity, physical activity, fatigue, young people, adolescents

## Abstract

**Highlights:**

**What are the main findings?**

- Paediatric post-COVID-19 condition patients showed reduced exercise capacity and muscle strength compared with healthy peers.
- Quadriceps echo-intensity and fatigue were increased, and school/sport limitations were frequent.

**What is the implication of the main finding?**

- Objective tests can assess and monitor paediatric post-COVID-19 conditions.
- Early detection supports targeted rehabilitation and interventions.

**Abstract:**

Background/Objectives: Lack of objective evidence exists regarding changes in physical function and impact on daily functioning in paediatric post-COVID-19 condition (PPCC). This study aimed to assess exercise capacity, fatigue, and peripheral and respiratory muscle strength in PPCC patients compared with healthy controls. Additionally, the impact of PPCC on domains of daily life was evaluated. Methods: A cross-sectional study was performed. Study variables: exercise capacity (6 min walk test, 6MWT), inspiratory muscle strength (maximal inspiratory pressure, PImax), handgrip strength (handheld dynamometer, HHD), quadriceps femoris muscle thickness (QF MT), rectus femoris muscle thickness (RF MT), rectus femoris cross-sectional area (RF CSA), rectus femoris echo-intensity (RF EI), fatigue (Paediatric Functional Assessment of Chronic Illness Therapy-Fatigue, pedsFACIT-F), and physical activity (Assessment of Physical Activity Levels Questionnaire, APALQ). Results: A total of 115 PPCC patients and 227 healthy controls were included. The PPCC group had lower 6MWT (509.00 ± 86.12, *p* < 0.001), PImax (68.71 ± 26.23, *p* < 0.001), HHD (82.84 ± 29.09, *p* < 0.001), APALQ (7.94 ± 3.14, *p* < 0.001), pedsFACIT-F (24.51 ± 11.01, *p* < 0.001), QF MT mid-thigh (33.21 ± 7.99, *p* = 0.011), and higher RF EI (*p* < 0.001) vs. controls. Only 37.63% of the PPCC group resumed previous sports, 43.48% were unable to attend school full-time and 28.7% could not participate in after-school activities. Conclusions: Paediatric post-COVID-19 condition patients exhibited significant impairments in terms of physical function, with a high impact on daily functioning. This knowledge is necessary to provide targeted therapeutic interventions.

## 1. Introduction

COVID-19 infection presents a wide spectrum of manifestations, ranging from asymptomatic cases to severe acute respiratory distress syndrome and death, involving multiple organ systems. Although severe COVID-19 is less common in children than in adults, paediatric patients can still be significantly affected and may develop long-term symptoms.

The paediatric post-COVID-19 condition (PPCC) was defined by the World Health Organization (WHO) in 2022 [1] as the presence of persisting symptoms in children and adolescents, lasting at least 2 months, which initially occurred within 3 months of confirmed or probable SARS-CoV-2 infection, that cannot be explained by an alternative diagnosis. Symptoms generally have an impact on everyday functioning and may fluctuate or relapse over time.

This cluster of symptoms is heterogeneous and can vary over time and with age. While emerging evidence suggests fatigue, weakness, and exertional dyspnoea are frequent in PPCC, objective data remain scarce, and many studies lack control groups or rely predominantly on self-reported outcomes [2,3,4,5].

A multidisciplinary unit was established at Germans Trias i Pujol University Hospital to evaluate and manage children and adolescents with PPCC. This unit was designed to offer a comprehensive, patient-centred and holistic approach. To provide the best management, it is necessary to first identify the specific dysfunctions affecting these patients.

This study aimed to identify research gaps and guide future investigations into the effects of post-COVID-19 condition on pediatric physical function. We hypothesised that children with PPCC would show reduced 6MWT distance, lower PImax and handgrip strength, higher fatigue, and reduced participation in school/sport versus healthy peers.

## 2. Materials and Methods

### 2.1. Study Design

This was an observational, analytical, cross-sectional study with an approximate 2:1 control-to-PPCC ratio. It was conducted at the ‘Pediatric Long COVID Multidisciplinary Unit’ of the tertiary care Germans Trias i Pujol University Hospital (Badalona, Barcelona, Spain). The study involved a multidisciplinary team comprising rehabilitation physicians, physiotherapists, paediatric practitioners, and specialists in infectious diseases, immunology, neurology, pulmonology, cardiology, gastroenterology, psychiatry, psychology, and radiology.

Children and young people affected by the post-COVID-19 condition (PPCC group), aged 8 to 17 years and meeting the inclusion criteria, were enrolled between 17 December 2020 and the data cut-off was 12 June 2024. The participants were referred from primary care centres, other hospitals in Catalonia, the Adult Long COVID Unit of our hospital, and the Catalan Long COVID Patient Association. During the same period, children and young people not affected by the post-COVID-19 condition were recruited as part of the control group, which included participants from a school (Escolapies School, El Masnou, Barcelona, Spain) and a sports club (Viladecans Baseball Club, Viladecans, Barcelona, Spain), all aged 8 to 17 years. Neither evaluators nor analysts were blinded to group allocation.

This study adhered to the Strengthening the Reporting of Observational Studies in Epidemiology (STROBE) reporting guidelines.

### 2.2. Legal and Ethical Considerations

This study was reviewed and approved by the Spanish Research Ethics Committee PI-21-029 CDC and PI-21-324 CDC (CEI-CEIC) and was conducted in full compliance with the Declaration of Helsinki, applicable local laws, and regulations. Informed consent was obtained from the participants’ parents or legal guardians prior to their enrolment.

The treatment, communication, and transfer of personal data were managed in accordance with the provisions of Regulation (EU) 2016/679 of the European Parliament and of the Council of 27 April 2016 on the General Data Protection Regulation (RGPD), which is applicable as of 25 May 2018.

### 2.3. Participants and Setting

Eligible participants in the PPCC group were children and young people aged 8 to 17 years who were referred to our unit. The inclusion criteria for participants were as follows: (i) exhibited three or more symptoms compatible with PPCC lasting longer than 12 weeks after SARS-CoV-2 infection that could not be explained by an alternative diagnosis, regardless of prior hospitalization, selected from the following checklist: fatigue, neurocognitive disorders, headache, muscular weakness, dyspnoea, myalgias/arthralgias, insomnia and other sleep disturbances, palpitations/tachycardia, chest oppressions/pain, orthostatic hypotension, loss of appetite/anorexia, deafness/tinnitus/phonophobia, photophobia, anosmia, ageusia/dysgeusia, abdominal pains, diarrhea, paresthesia, dizziness/vertigo, cough, persistent fever/chills, skin signs, epigastric pains/dyspepsia/food impact, odynophagia/dysphagia, vomiting/nauseas, dysphonia and rhinorrhea; (ii) had a confirmed SARS-CoV-2 infection diagnosis or clinical suspicion (i.e., COVID-19 symptoms in participants with a close relative with confirmed infection during community transmission at the beginning of the pandemic, March 2020, when testing was inaccessible); (iii) presented persisting symptoms absent before the infection; and (iv) presented persistent symptoms impacting daily functioning. Participants were excluded if they were unable to provide informed consent, failed to attend follow-up visits, or had psychiatric or cognitive impairments preventing them from completing evaluations. Participants with coexisting musculoskeletal or neurological conditions limiting mobility were also excluded.

Eligible participants in the control group were children and young people aged 8 to 17 years who were voluntarily recruited from a school and a sports club. Parents or legal guardians of controls who were eligible for inclusion signed informed consent forms. For controls, prior SARS-CoV-2 infection was assessed by a self-report questionnaire, as the study objective was to compare children with and without persistent symptoms. The exclusion criteria included persistent symptoms longer than 12 weeks compatible with a PPCC diagnosis, inability to provide informed consent, psychiatric or cognitive impairments interfering with evaluations, and coexisting musculoskeletal or neurological conditions affecting mobility.

### 2.4. Study Procedures and Data Collection

The research team reviewed the medical records of participants at our unit. Data for the PPCC group were collected during outpatient visits using standardized forms from electronic medical records, anamnesis, physical examinations, and questionnaires. These assessments included evaluations of fatigue and physical activity, physical function through exercise capacity, respiratory and upper limb muscle strength, and ultrasound measurements of the quadriceps femoris. Two scientific advisors reviewed all the collected data. The information recorded included demographic details; medical and family history; SARS-CoV-2 infection data; symptoms during the acute COVID-19 phase; and persistent symptoms, such as postural tachycardia syndrome, orthostatic hypotension, fatigue, weakness, arthralgia, neurocognitive impairments, and respiratory, cardiological, and gastrointestinal symptoms. For PPCC patients without microbiological confirmation of SARS-CoV-2 infection by PCR, antigen testing or serology, an immune functional study (SARS-CoV-2-specific T-cell immunity assay) was performed.

Data for the control group were collected onsite at the school or sports club using the same standardized forms, which included anamnesis, physical examinations, questionnaires assessing fatigue and physical activity, exercise capacity evaluations, measurements of respiratory and upper limb muscle strength, and ultrasound evaluations of the quadriceps femoris. Two scientific advisors reviewed these data, which also included demographic information, medical history, prior COVID-19 infection history and anthropometric measures (weight, height, body mass index (BMI), blood pressure, heart rate, respiratory rate, and arterial oxygen saturation).

#### 2.4.1. Physical Function Assessments

Exercise capacity was evaluated using the 6 min walk test (6MWT) [6]. Participants were instructed to walk as fast as possible along a flat, hard surface between two cones positioned 24 m apart, and the maximum distance (in metres) walked in 6 min was recorded. Dyspnoea and fatigue perception were assessed using the Borg scale (0–10, where 0 indicates no dyspnoea/fatigue and 10 maximal dyspnoea/fatigue) [7], immediately before and after the test.

Handgrip strength was measured using a handheld dynamometer (Jamar^®^ Hydraulic Hand Dynamometer, Madrid, Spain) in both hands following a standardized protocol [8]. For each hand, the maximum value of three attempts was recorded in kilograms (absolute handgrip strength), also noting hand dominance. Absolute values were then compared with age-, sex- and hand-specific reference values for the Spanish paediatric population [9], and expressed as percentage of predicted (% predicted). Since handgrip strength is influenced by sex, age and hand dominance, percentage values (% predicted) were considered the most reliable measure to assess handgrip strength [10].

Respiratory muscle strength, specifically maximal inspiratory pressure (PImax), was measured using a digital mouth pressure meter MicroRPM (MicroMedical-Carefusion, Madrid, Spain) in accordance with American Thoracic Society/European Respiratory Society (ATS/ERS) guidelines [11]. The Participants remained in the sitting position with trunk erect, hip and knee flexed to 90 degrees and foot placed on the ground. Before testing, two maximal inspiratory maneuvers were performed for familiarization. A nose clip was used in all maneuvers. For PImax measurement, participants were instructed to perform a maximal inspiratory effort starting from the residual volume (RV), and the values were recorded. The highest value of three reproducible maneuvers (<10% variability between values) was recorded. Participants were allowed to rest one minute between each trial maneuver. Reference values for paediatric populations reported by Szeinberg et al. [12], expressed in cmH_2_O according to age and sex, were used to establish the lower limit of normal (LLN). Each participant’s PImax was then compared with these reference values and classified as below LLN, within the normal range, or not evaluable.

A portable ultrasound device (VINNO 5) was used to obtain ultrasound images of both thigh muscles, including rectus femoris (RF) and quadriceps femoris (QF). B-mode transverse images were obtained with a high-frequency (7.5 MHz) linear transducer by a single investigator. The participants lay supine with their hips in a neutral position and knees fully extended and relaxed. The landmark was taken from the distance between the anterosuperior iliac spine (ASIS) to the superior border of patella (SBP), bilaterally and noting dominance [13]. Two sites were marked with a skin pen where images were taken: the mid-thigh and distal thigh, which were 1/2 and 2/3, respectively, of the distance from the ASIS to SBP. Ultrasound gel was applied, and the transducer was positioned perpendicular to the skin surface with minimal pressure to prevent tissue distortion. The measurements included quadriceps femoris muscle thickness (QF MT), rectus femoris muscle thickness (RF MT) and rectus femoris cross-sectional area (RF CSA). RF MT was defined in millimetres from the superficial fascia to deep fascia of rectus femoris. QF MT was defined as the combined thicknesses of RF and the vastus intermedius (VI), in millimetres, from the superficial fascia of the RF to the bone surface. RF MT and QF MT were measured at mid-thigh and distal thigh (1/2 and 2/3 sites, respectively). RF CSA was measured in centimetres at 2/3 site, the only place where its complete measurement is allowed in a single image. The mean value of 2 measurements was used in the analysis. Intra-observer reliability of muscle ultrasound measurements (muscle thickness and cross-sectional area) was assessed using the intraclass correlation coefficient (ICC) with 95% confidence intervals, based on two repeated measurements. In addition, echo-intensity of rectus femoris (RF EI) of the dominant limb, assessed at the 2/3 site, was categorized using the Heckmatt rating scale [14]: (I) normal echogenicity, (II) increased muscle echogenicity with normal bone reflection, (III) increased muscle echogenicity with reduced bone reflection, and (IV) markedly increased muscle echogenicity with loss of bone reflection.

#### 2.4.2. Physical Activity and Fatigue Assessments

Physical activity was assessed using the International Assessment of Physical Activity Levels Questionnaire (APALQ) for children and young people [15,16]. Scores range from 5 to 22, with higher values indicating greater physical activity. Physical activity was classified as sedentary (≤10), moderately active (11–16), or very active (≥17).

Fatigue was evaluated using the Pediatric Functional Assessment of Chronic Illness Therapy—Fatigue (pedsFACIT-F) scale, a 13-item questionnaire designed for children and young people aged 8–18 years [17,18,19]. This tool measures self-reported fatigue and its impact on daily activities and functions. Scores range from 0 to 52, with higher scores reflecting less fatigue. Categories were defined as follows: ≥45, fatigue-free; 31–44, low fatigue; 21–30, moderate fatigue; 11–20, high fatigue; and 0–10, very high fatigue.

#### 2.4.3. School and Activity Participation

Information on school attendance, sports participation, and after-school activities was collected using a self-reported questionnaire completed by participants. Variables included regular participation in sport/physical activity (yes/no); ability to continue the same activity as before infection (yes/no); regular weekly hours of sport/physical activity (0, 0–2, 2–4, 4–6, >6); and current weekly hours of sport/physical activity. School attendance was categorised as full, partial, or unable to attend, and after-school activities as full attendance, unable to attend, or not applicable/none.

### 2.5. Statistical Analysis

The fatigue rate has been calculated in a cohort of paediatric patients with long COVID with 10% precision. A random sample of 115 individuals is sufficient to calculate, with a 95% confidence level and 9% accuracy, a population fatigue rate expected to be approximately 60% in patients with long COVID. The sample size calculation accounts for a 5% loss rate. To explore the differences between cases and controls, a control group with a 2:1 ratio was recruited.

The baseline characteristics are summarized in the tables and were categorized by study group (PPCC group, school control group and sports club control group). Categorical variables are reported as frequencies and percentages of each category, whereas continuous variables are expressed as the means and standard deviations. Differences in outcome variables between the PPCC group and control groups at baseline were analysed by calculating Cohen’s d and 95% confidence intervals and performing *t* tests or Wilcoxon-Mann–Whitney, depending on the distribution of the variable. Linear models with age and sex as adjustment variables were used to examine the associations between ECO parameters and age at baseline. Models were calculated separately for PPCC and controls to examine the differential effects of the interaction between age and sex on ECO parameters in both groups. Variable collinearity was assessed in all models using the variance inflation factor.

Model assumptions were validated, and 95% confidence intervals were computed wherever applicable. All the statistical analyses were conducted using the R software (version 4.4.0, released 14 June 2024) for Windows.

## 3. Results

### 3.1. Characteristics of the Study Sample

Table 1 summarizes the demographic, clinical, anthropometric, and activity-related characteristics of the study groups (PPCC, school controls, and sports club controls).

At recruitment, the mean age was 13.31 years (±2.25; range, 8–17 years) in the PPCC group, 11.01 years (±2.21) in the school controls, and 12.33 years (±2.5) in the sports club controls. Females predominated in the PPCC group (66.09%), compared with 47.73% in the school controls and 52.94% in the sports club controls.

Most participants were within the normal weight range (81.74% in PPCC, 93.75% in school controls, and 84.31% in sports club controls). However, obesity was more frequent in the PPCC group (11.30%) than in the school (5.11%) and sports club (7.84%) controls. Background medical conditions are described in Table A1.

Before COVID-19 infection, regular sport or physical activity was reported by 80.87% of PPCC participants, 82.95% of school controls, and all sports club controls. After infection, 62.37% of the PPCC group were unable to return to their previous activity levels, with only 37.63% resuming the same activities.

Regarding school attendance, 43.48% of the PPCC group faced significant limitations: 31.30% attended only part-time, and 12.17% were unable to attend classes. Furthermore, 44% of the PPC group could not participate in after-school activities.

### 3.2. Clinical Characteristics

As physical function could be associated with being sporty or not, PPCC were first compared separately with school controls and sports club controls (Table A2). No significant differences were detected in either comparison; therefore, the two control groups were analysed together in subsequent analyses.

Analysis of physical function variables (Table 2) revealed that exercise capacity (6MWT), dyspnoea and fatigue perception before and after the 6MWT, and physical activity (APALQ) were significantly lower in the PPCC group compared with controls (all *p* < 0.001). Regarding physical activity categories, 77.39% of PPCC participants were classified as sedentary (*n* = 89), whereas only 13.22% of controls fell into this category. Conversely, 20.87% of the PPCC group were moderately active compared with 81.5% of the controls.

In terms of peripheral muscle strength, PPCC group showed significantly lower values of handgrip strength (% predicted) in both hands compared with controls (*p* < 0.001, Table 2). Respiratory muscle strength, measured by maximal inspiratory pressure (PImax), was also significantly lower in the PPCC group (*p* < 0.001, Table 2). However, when PImax was analysed against paediatric reference values reported by Szeinberg et al., no significant differences were observed (*p* = 0.796), probably because of the large number of nonvaluable subjects (*n* = 104).

Analysis of ultrasound variables (Table 2) revealed significant differences in rectus femoris echo-intensity (RF EI). Ultrasound measurements showed good to excellent reliability (ICC 0.81–0.98; Table A3). Although grade I was the predominant classification in both groups (PPCC: 86/106, 81.1%; controls: 224/227, 98.7%), the distribution differed significantly (*p* < 0.001). In the PPCC group, 19 participants (19.9%) were classified as grade II, compared with only 2 participants (0.9%) in the control group. Regarding muscle thickness, quadriceps femoris muscle thickness (QF MT; sum of rectus femoris and vastus intermedius) at mid-thigh was significantly lower in the PPCC group than in controls. Since differences in thickness were observed only at mid-thigh and exclusively for QF, we considered the possibility of confounding by age and sex. Although these potential biases cannot be confirmed due to the cross-sectional design, additional analyses suggested a differential influence of age and sex on muscle thickness and cross-sectional area in controls, which was not observed in the PPCC group.

With respect to fatigue (Table 2), almost all PPCC participants (110/115; 95.67%) reported fatigue, whereas only 1/227 controls (0.4%) did so (*p* < 0.001). Within the PPC group, 29.57% reported low fatigue, 26.96% moderate, 29.57% high, and 9.57% very high fatigue. In turn, the PPCC group had a significantly higher body mass index than controls (21.33 vs. 19.33 kg/m^2^, *p* < 0.001).

## 4. Discussion

This was a cross-sectional study of paediatric patients between 8 and 17 years of age who were referred to the ‘Paediatric Long COVID Multidisciplinary Unit’ of a tertiary care hospital in Catalonia, compared with a control group of children and adolescents without persistent symptoms. The most common symptoms or impairments requiring rehabilitation in patients with PPCC include fatigue, weakness, dyspnoea and exercise intolerance. There has been a lack of research regarding objective findings of these symptoms and, therefore, regarding treatment [20,21]. Our aim was therefore to quantify these dysfunctions through validated questionnaires and standardized tests to provide appropriate education for families, improve symptom management, and guide rehabilitation programs with specific goals.

Several studies have described fatigue, exercise intolerance and weakness as common disturbances in PPCC [22,23,24]. In an Italian survey of 510 children, Buonsenso et al. [25] found fatigue and reduced activity in most cases, but their study lacked controls, relied on non-validated online questionnaires, and lacked objective assessments. Similarly to the findings of Buonsenso, in our cohort, children with PPCC reported greater fatigue and lower activity level than controls, with over half showing moderate to severe fatigue on the pedsFACIT-F scale. In contrast, our study combines a contemporaneous control group with validated instruments and in-person clinical assessments, allowing for a more reliable and clinically meaningful comparison.

Many studies overlook PPCC’s daily functioning, focusing solely on symptom persistence, making it difficult to assess the effect of symptoms on daily functioning. One notable exception is the LongCOVIDKidsDK study by Berg et al. [26], which demonstrated this impact through validated tools, such as the Pediatric Quality of Life Inventory (PedsQL) and the Children’s Somatic Symptom Inventory-24 (CSSI-24), and stands out for its large sample size and the inclusion of a matched control group. However, data were collected exclusively through online questionnaires without any face-to-face assessment. By contrast, our study integrates validated patient-reported measures (APALQ, pedsFACIT-F) with objective clinical assessments (6MWT, PImax, handgrip strength, QF muscle thickness and echo-intensity), providing a more comprehensive and clinically robust characterization of PPCC.

When comparing our work with other hospital-based cohorts that also evaluated PPCC in person, some methodological strengths of our study become clear. Regarding exercise capacity, Garai et al. [27] and Dobkin et al. [28] reported smaller series without control groups, and the 6MWT was only performed in selected participants (29 and 9, respectively), while we applied it systematically to all 115 PPCC. Dobkin et al. also assessed respiratory muscle strength, but this was limited to 7 children, while in our study, PImax was measured consistently across the entire cohort.

The pathophysiology is not well-understood. In adults, persistent respiratory symptoms have been linked to fibrotic changes, but such alterations have not been demonstrated in children [29]. In our cohort, lower PImax values compared with controls suggest inspiratory muscle weakness, most likely involving the diaphragm, which may explain exertional dyspnoea in the absence of pulmonary or cardiac disease. These findings support the hypothesis that persistent symptoms of PPCC, including fatigue, dyspnoea, and exercise intolerance, may be related to vagus nerve dysfunction, as recently proposed in adults [30].

We also found reduced handgrip strength, decreased quadriceps muscle thickness at mid-thigh only, and higher echo-intensity values in PPCC patients. Elevated echo-intensity indicates greater intramuscular infiltration by non-contractile elements such as fat or fibrotic tissue and is considered a reliable marker of muscle quality, with higher values associated with reduced functional capacity and muscle strength in clinical and aging populations [31]. However, this parameter has not been studied in PPCC before and deserves further investigation. Interestingly, in subsequent analyses of muscle thickness, thickness increased with age and male sex, reflecting normal developmental trends, whereas this pattern was absent in the PPCC group, suggesting that the post-COVID-19 condition may interfere with expected growth trajectories, a finding that should be explored longitudinally.

Our study has several limitations. It was conducted in a single centre and included tertiary referrals, which may have introduced selection bias towards more severe cases. Pre-COVID baseline data on muscle function were not available, preventing comparison with individual pre-infection status. In controls, infection history was self-reported, which may have caused misclassification; however, this was considered acceptable given the high prevalence of unnoticed infections in children and our focus on comparing symptomatic with asymptomatic peers. The cross-sectional design also limits causal inference and prevents assessment of symptom evolution over time. As some controls were recruited from a sports club, selection bias cannot be entirely excluded, although subsequent analyses showed no significant differences compared with non-athletic controls. Finally, no randomized control group was included, as such a design was considered unethical in the absence of clinical equipoise, in line with guidance at the time of recruitment.

## 5. Conclusions

Our study shows that PPCC patients exhibit reduced exercise capacity, lower inspiratory and peripheral muscle strength, and greater fatigue when compared with healthy controls. Ultrasound of the quadriceps femoris also revealed greater echogenicity, a finding consistent with impaired muscle quality. These abnormalities translate into practical limitations in daily functioning and illustrate the burden of PPCC during a vulnerable stage of growth and development.

Some children show a tendency to recover on their own, but in many others, the difficulties remain over time. This ongoing impairment underlines the importance of detecting the post-COVID-19 condition early and coordinating care through a multidisciplinary team. Rehabilitation programs that combine aerobic exercise, muscle strength and endurance, and inspiratory muscle training adapted to each child, along with education and strategies for symptom management, should be considered part of routine care.

Using validated assessment tools and clinical evaluations, our study shows that children and adolescents with post-COVID-19 condition experience measurable functional limitations. These findings lay the groundwork for developing tailored rehabilitation strategies and structured care models. Further follow-up studies are necessary to understand the significance of these impairments and the trajectory of persistent symptoms. Finally, clinical trials are required to evaluate the effectiveness of rehabilitation programs in symptom recovery and improvements in patients’ quality of life.

## Figures and Tables

**Table 1:** Demographic and activity-related characteristics across study groups (PPCC, school controls, and sports club controls) (N = 342).

Variables	PPCC Group N = 115	School Control Group N = 176	Sports Club Control Group N = 51
Sex			
Female	76 (66.09)	84 (47.73)	27 (52.94)
Male	39 (33.91)	92 (52.27)	24 (47.06)
Age, *n* (years)	13.31 ± 2.25	11.01 ± 2.21	12.33 ± 2.50
Positive diagnostic test for SARS-CoV-2			
No ^b^	2 (1.74)	63 (35.8)	26 (50.98)
Yes	113 (98.26)	113 (64.2)	25 (49.02)
Background medical conditions			
No	38 (33.04)	143 (81.25)	43 (84.31)
Yes	77 (66.96)	33 (18.75)	8 (15.69)
BMI, kg/m^2^	21.33 ± 4.50	18.62 ± 3.33	20.94 ± 3.78
Weight percentile	51.70 ± 31.46	40.99 ± 25.25	54.69 ± 28.49
Healthy weight by percentile	94 (81.74)	165 (93.75)	43 (84.31)
Overweight by percentile (>90)	8 (6.96)	2 (1.14)	4 (7.84)
Obesity by percentile (>97)	13 (11.30)	9 (5.11)	4 (7.84)
Regular sport/physical activity			
No	22 (19.13)	30 (17.05)	0 (0)
Yes	93 (80.87)	146 (82.95)	51 (100)
If you practice regularly, could you practice the same sport/physical activity right now?			
No	58 (62.37)	0 (0)	0 (0)
Yes	35 (37.63)	146 (100)	51 (100)
Regular weekly hours of sport/physical activity	4.00 ± 3.92	3.64 ± 2.9	6.08 ± 2.13
0 h	21 (18.26)	30 (17.05)	0 (0)
0–2 h	24 (20.87)	47 (26.7)	0 (0)
2–4 h	30 (26.09)	20 (11.36)	0 (0)
4–6 h	16 (13.91)	52 (29.55)	40 (78.43)
>6 h	24 (20.87)	27 (15.34)	11 (21.57)
Current weekly hours of sport/physical activity	0.92 ± 1.6		
After-school activities: full attendance	42 (36.52)	131 (74.43)	39 (76.47)
After-school activities: unable to attend	33 (28.7)	0 (0)	0 (0)
After-school activities: not applicable/none	40 (34.78)	45 (25.57)	12 (23.53)
School attendance: full	65 (56.52)	176 (0)	51 (0)
School attendance: partial	36 (31.3)	0 (0)	0 (0)
School attendance: unable to attend	14 (12.17)	0 (0)	0 (0)

Abbreviations: SARS-CoV-2: severe acute respiratory syndrome coronavirus 2; BMI: body mass index; PPCC: paediatric post-COVID-19 condition. Notes: ^b^ No microbiological/immunological confirmation: COVID-19 symptoms in participants with a close relative who had confirmation of SARS-CoV-2 infection in a situation of community transmission at the beginning of the pandemic, when it was not possible to access a test. Data are presented as the mean ± SD or frequency (percentage).

**Table 2:** Comparison of physical function, fatigue and activity outcomes between PPCC and controls (N = 342).

Variables	PPCC N = 115	Controls N = 227	Difference	95% CI	*p* Value
6MWT, m	509.00 ± 86.12	666.47 ± 82.56	−1.88	−2.15, −1.61	<0.001
Dyspnoea (Borg 0–10), pre-6MWT	1.19 ± 1.69	0.00 ± 0.00	1.24	0.99, 1.48	<0.001
Dyspnoea (Borg 0–10), post-6MWT	4.35 ± 2.56	1.64 ± 1.25	1.51	1.26, 1.77	<0.001
Fatigue (Borg 0–10), pre-6MWT	2.39 ± 2.46	0.04 ± 0.28	1.65	1.39, 1.91	<0.001
Fatigue (Borg 0–10), post-6MWT	5.20 ± 2.88	2.89 ± 2.02	0.99	0.75, 1.23	<0.001
Handgrip (D), kg	20.08 ± 8.63	21.49 ± 9.34	−0.16	−0.38, 0.07	0.168
Handgrip, % predicted (D)	82.84 ± 29.09	107.79 ± 26.50	−0.91	−1.15, −0.67	<0.001
Handgrip (ND), kg	18.83 ± 8.47	20.03 ± 9.32	−0.13	−0.36, 0.09	0.236
Handgrip, % predicted (ND)	82.10 ± 30.46	106.15 ± 28.2	−0.83	−1.06, −0.6	<0.001
PImax, cmH_2_O	68.71 ± 26.23	94.18 ± 25.91	−0.98	−1.27, −0.69	<0.001
Reference LLN PImax (Szeinberg), cmH_2_O	95.09 ± 11.37	95.51 ± 11.78	−0.04	−0.31, 0.24	0.796
Bellow LLN by Szeinberg	50 (73.53)	2 (0.89)			
Within normal range by Szeinberg	8 (11.76)	129 (57.33)			
Not evaluable by Szeinberg	10 (14.71)	94 (41.78)			
QF MT mid-thigh (D), mm	33.21 ± 7.99	30.91 ± 6.69	0.32	0.09, 0.55	0.011
RF MT mid-thigh (D), mm	17.96 ± 3.71	17.44 ± 3.79	0.14	−0.09, 0.37	0.243
QF MT distal thigh (D), mm	24.89 ± 6.64	23.86 ± 5.36	0.18	−0.05, 0.41	0.165
RF MT distal thigh (D), mm	12.67 ± 3.66	12.38 ± 3.01	0.09	−0.14, 0.32	0.480
RF CSA (D), cm^2^	3.61 ± 1.35	3.36 ± 1.26	0.2	−0.04, 0.43	0.107
QF MT mid-thigh (ND), mm	32.54 ± 7.38	30.37 ± 6.56	0.32	0.08, 0.55	0.011
RF MT mid-thigh (ND), mm	17.61 ± 3.52	17.04 ± 3.52	0.16	−0.07, 0.39	0.165
QF MT distal thigh (ND), mm	24.10 ± 6.28	23.63 ± 5.32	0.08	−0.15, 0.31	0.512
RF MT distal thigh (ND), mm	12.12 ± 3.42	12.15 ± 2.86	−0.01	−0.24, 0.22	0.948
RF CSA (ND), cm^2^	3.42 ± 1.28	3.28 ± 1.16	0.12	−0.11, 0.35	0.327
RF EI					<0.001
RF EI I	86 (81.13)	224 (98.68)			
RF EI II	19 (17.92)	2 (0.88)			
RF EI III	1 (0.94)	1 (0.44)			
RF EI IV	0 (0)	0 (0)			
pedsFACIT-F	24.51 ± 11.01	50.08 ± 1.78	−3.91	−4.28, −3.54	<0.001
pedsFACIT-F categories					
Fatigue-free (45–52 score)	5 (4.35)	226 (99.56)			
Low (31–44 score)	34 (29.57)	1 (0.44)			
Moderate (21–30 score)	31 (26.96)	0 (0)			
High (11–20 score)	34 (29.57)	0 (0)			
Very high (0–10 score)	11 (9.57)	0 (0)			
Weight percentile	51.70 ± 31.46	44.07 ± 26.57	0.27	0.04, 0.49	0.027
BMI, kg/m^2^	21.33 ± 4.5	19.13 ± 3.56	0.56	0.34, 0.79	<0.001
APALQ	7.94 ± 3.14	12.92 ± 2.82	−1.7	−1.96, −1.44	<0.001
APALQ categories					
Sedentary (5–10)	89 (77.39)	30 (13.22)			
Moderately active (11–16)	24 (20.87)	185 (81.5)			
Very active (>17)	2 (1.74)	12 (5.29)			

Abbreviations: 95% CI: 95% confidence interval; APALQ: Assessment of Physical Activity Levels Questionnaire; pedsFACIT-F: Pediatric Functional Assessment of Chronic Illness Therapy-Fatigue; 6MWT: 6 min walk test; PImax: maximal inspiratory pressure; LLN: lower limit of normal; reference values in cmH_2_O according to age and sex from Szeinberg et al. [12]; classifications “bellow LLN”, “within normal range”, and “not evaluable” were based on these reference values; QF MT: quadriceps femoris muscle thickness; RF MT: rectus femoris muscle thickness; RF CSA: cross-sectional area; RF EI: rectus femoris echo-intensity, graded I-IV according to the Heckmatt scale [14], (I: normal echogenicity; IV: markedly increased echogenicity with loss of bone reflection); Borg: Borg scale (0–10); pre: immediately before the 6MWT; post: immediately after the 6MWT. BMI: Body mass index; D: Dominant; ND: non-dominant; PPCC: paediatric post-COVID-19 condition. Notes: Data are presented as the mean ± SD or frequency (percentage). Difference and 95% CI were calculated using Cohen’s D. *p*-values were calculated using Wilcoxon Mann–Whitney test (dyspnoea and fatigue, Borg scale), Fisher’s exact test (RF EI categories) and *t*-test (all other variables).

## Data Availability

The raw data supporting the conclusions of this article will be made available by the authors upon request.

## Appendix A

**Table A1:** Background medical conditions of the study sample (N = 342).

	PPCC Group N = 115	School Control Group N = 176	Sports Club Control Group N = 51
Respiratory system disorders			
Asthma	12 (10.43)	8 (4.55)	3 (5.88)
Bronchitis	11 (9.57)	10 (5.68)	1 (1.96)
Alpha-1 antitrypsin deficiency		1 (0.57)	
Recurrent pneumonia in childhood	2 (1.74)	5 (2.84)	
Bronchiolitis in childhood	3 (2.61)		
Cardiovascular disorders			
Wolff Parkinson white syndrome ^b^	1 (0.87)		
Interatrial communication	1 (0.87)		
Neurological-development disorders			
Cognitive developmental delay		1 (0.57)	
Migraine	9 (7.83)		
Postinfectious cerebellitis ^b^	1 (0.87)		
Duane syndrome	1 (0.87)		
Recurrent nonspecific headache	8 (6.96)		
Dyslexia	4 (3.48)		
Autism spectrum disorder (level 1)	3 (2.61)		
Psychiatric disorders			
Depression	5 (4.35)		
Attention deficit hyperactivity disorder	9 (7.83)	1 (0.57)	
Anxiety	5 (4.35)		1 (1.96)
Adjustment disorder	1 (0.87)		
Autoimmune system disorders			
Lupus erythematosus	1 (0.87)		
Autoimmune hypothyroidism	2 (1.74)		
Celiac disease	3 (2.61)		
Traumatological disorders			
Femoral osteochondroma		1 (0.57)	
Osgood Schlatter disease	1 (0.87)		
Scoliosis	3 (2.61)		
Sever’s disease ^b^	1 (0.87)		
Otovestibular disorders			
Peripheral vertigo	2 (1.74)		
Labyrinthitis	1 (0.87)		
Conductive hearing loss	2 (1.74)		
Endocrine–metabolic disorders			
Obesity	2 (1.74)		
Diabetes mellitus 1		1 (0.57)	
Subclinical hypothyroidism	1 (0.87)		
Short stature ^b^	2 (1.74)		
Gastrointestinal disorders			
*Helicobacter pylori* infection ^b^	2 (1.74)		
Mesenteric adenitis	1 (0.87)		
Oncology disorders			
Leukaemia ^b^		1 (0.57)	
Allergic disorders			
Atopic dermatitis	22 (19.13)	8 (4.55)	4 (7.84)
Rhinoconjunctivitis	10 (8.70)		
Chronic urticaria	2 (1.74)		
Other Disorders			
Chronic fatigue	1 (0.87)		
Heterozygous mutation of factor V Leiden	1 (0.87)		
Haemochromatosis	1 (0.87)		
Chromosome 3q29 microdeletion	1 (0.87)		
Polydactyly	1 (0.87)		
Vitíligo	1 (0.87)		
Dengue ^b^	1 (0.87)		
Hereditary angioedema	1 (0.87)		

Abbreviations: PPCC: paediatric post-COVID-19 condition. Notes: ^b^ These medical conditions were resolved during the present study. Data are presented as frequency (percentage).

**Table A2:** Comparison of exercise capacity, fatigue, dyspnoea, and physical activity between PPCC and control groups (N = 342).

Variables	PPCC Group N = 115	School Control Group N = 176	Difference	95% CI	*p* Value	Sports Club Control Group N = 51	Difference	95%CI	*p* Value
6MWT, m	509.00 ± 86.12	677.60 ± 86.36	−1.95	−2.24, −1.67	<0.001	628.06 ± 52.58	−1.54	−1.91, −1.17	<0.001
Dyspnoea (Borg 0–10), pre-6MWT	1.19 ± 1.69	0.00 ± 0.00	1.14	0.88, 1.39	<0.001	0.00 ± 0.00	0.85	0.51, 1.2	<0.001
Dyspnoea (Borg 0–10), post-6MWT	4.35 ± 2.56	1.78 ± 1.13	1.41	1.15, 1.68	<0.001	1.16 ± 1.53	1.4	1.03, 1.76	<0.001
Fatigue (Borg 0–10), pre-6MWT	2.39 ± 2.46	0.00 ± 0.00	1.57	1.29, 1.84	<0.001	0.16 ± 0.58	1.08	0.73, 1.43	<0.001
Fatigue (Borg 0–10), post-6MWT	5.20 ± 2.88	3.25 ± 1.96	0.82	0.58, 1.07	<0.001	1.65 ± 1.71	1.38	1.01, 1.74	<0.001
APALQ (score 5–22)	7.94 ± 3.14	12.46 ± 2.99	−1.48	−1.75, −1.22	<0.001	14.51 ± 1.17	−2.44	−2.86, −2.01	<0.001
APALQ categories									
Sedentary (5–10)	89 (77.39)	30 (17.05)				0 (0)			
Moderately active (11–16)	24 (20.87)	140 (79.55)				45 (88.24)			
Very active (>17)	2 (1.74)	6 (3.41)				6 (11.76)			

Abbreviations: 95% CI: 95% confidence interval; APALQ: Assessment of Physical Activity Levels Questionnaire; 6MWT: 6 min walk test; Borg: Borg scale (0–10); pre: immediately before the 6MWT; post: immediately after the 6MWT. PPCC: paediatric post-COVID-19 condition. Data are presented as the mean ± SD or frequency (percentage). Difference and 95% CI were calculated using Cohen’s D. *p*-values were calculated using Wilcoxon Mann–Whitney test (dyspnoea and fatigue, Borg scale), and *t*-test (all other variables).

**Table A3:** Intraclass correlation coefficients (ICC) for quadriceps and rectus femoris ultrasound measurements in PPCC and controls (N = 342).

Variables	ICC PPCC Group	ICC Control Group
QF MT mid-thigh (D), mm	0.961 [0.944;0.973]	0.936 [0.918;0.951]
RF MT mid-thigh (D), mm	0.878 [0.828;0.914]	0.88 [0.847;0.907]
QF MT distal thigh (D), mm	0.938 [0.912;0.957]	0.865 [0.828;0.894]
RF MT distal thigh (D), mm	0.867 [0.813;0.906]	0.831 [0.786;0.868]
RF CSA (D), cm^2^	0.929 [0.899;0.951]	0.928 [0.908;0.944]
QF MT mid-thigh (ND), mm	0.811 [0.738;0.866]	0.864 [0.827;0.894]
RF MT mid-thigh (ND), mm	0.897 [0.854;0.928]	0.906 [0.879;0.927]
QF MT distal thigh (ND), mm	0.821 [0.752;0.873]	0.841 [0.798;0.875]
RF MT distal thigh (ND), mm	0.983 [0.975;0.988]	0.985 [0.981;0.989]
RF CSA (ND), cm^2^	0.982 [0.974;0.987]	0.977 [0.97;0.982]

Abbreviations: PPCC: paediatric post-COVID-19 condition; QF MT: quadriceps femoris muscle thickness; RF MT: rectus femoris muscle thickness; RF CSA: cross-sectional area; D: Dominant; ND: non-dominant. ICC: intraclass correlation coefficient; CI: confidence interval. Values are ICC with 95% CI based on two repeated measurements.
